# Head and neck cancer rehabilitation: the CaRe feasibility study

**DOI:** 10.1007/s00520-026-10839-z

**Published:** 2026-06-04

**Authors:** Grainne Sheill, Sophie Grehan, Annemarie E. Bennett, Conor Bowe, Julie Broderick, Kelly Coghlan, Julie Regan, Juliette Hussey, John Ed O. Connell

**Affiliations:** 1 University of Dublin, Trinity College Dublin, Dublin, Ireland; 2 Trinity St James’s Cancer Institute, Dublin, Ireland; 3 National Oral and Maxillofacial Unit, St. James’ Hospital, Dublin, Ireland; 4 Physiotherapy Department, Hospital 4, St James’s Hospital, Dublin, Dublin 8 D08E9P6 Ireland

**Keywords:** Feasibility, Cancer rehabilitation, Head and neck cancer, Physiotherapy

## Abstract

**Purpose:**

The aim of this study was to evaluate the feasibility and acceptability of a multi-modal exercise and education rehabilitation programme for patients post head and neck cancer (HNC) treatment.

**Methods:**

This single-arm prospective feasibility study included patients in the first 2 years following treatment for HNC. Participants completed a 10-week multi-modal exercise and education programme including twice weekly online or in-person group exercise sessions and four education sessions. Feasibility was evaluated via recruitment, adherence, and compliance to the programme. Secondary outcomes examined physical function and quality of life. The acceptability of the programme was assessed through participant feedback.

**Results:**

In total, 22 participants were recruited (36% (*n* = 8) female, mean age 65.23 years (SD 14.53, range 27–87 years)). The recruitment rate was 24%. Most participants had a history of surgery including neck dissection (95.5%, *n* = 21) and seven had flap reconstruction (31.8%). Approximately 54.5% (*n* = 12) of the participants screened positively for lymphoedema. Five people engaged with classes online (average 16.25 classes, range 6–24). Most participants attended the in-person class (*n* = 14, average 9.86 classes range 1–20). One person chose to do online and in-person classes. Two participants enrolled in the online programme did not engage with the intervention. Measures of physical activity levels, strength, frailty, and physical well-being all increased significantly post intervention. Participants provided positive feedback on the programme, particularly valuing the social experience of exercising alongside peers with similar cancer experiences.

**Conclusions:**

The intervention appears feasible in a group of complex cancer survivors of HNC, with a preference for in-person exercise classes.

**ClinicalTrials.gov registration number:**

NCT06646861

**Supplementary Information:**

The online version contains supplementary material available at https://doi.org/10.1007/s00520-026-10839-z.

## Introduction

Head and neck cancer (HNC) encompasses malignancies that arise in the nasal cavity, oral cavity, pharynx, and larynx. The incidence of HNC is increasing worldwide, and nearly two-thirds of patients diagnosed with HNC will live for at least 5 years [1]. Most patients are treated with a combination of surgery, radiotherapy, and/or chemotherapy [2]. These treatments are associated with many unique survivorship issues such as dry mouth and throat, lymphoedema, cancer-related fatigue, shoulder weakness, and pain [3]. Numerous systematic reviews demonstrate that exercise can mitigate a number of these issues in cancer survivors [4, 5]. Exercise is also associated with improved quality of life, cardiorespiratory fitness, physical functioning, and functional status, preserving the ability of patients to remain in the workforce and fulfil other life roles [6–8]. Despite the well-documented benefits of engaging with physical activity during and after cancer, side effects of treatment for head and neck cancer can decrease people’s ability to engage in physical activity and result in poorer cardiorespiratory fitness [9–13].

The development of supportive interventions for patients in the acute phase post cancer treatment is needed [14], including personalised exercise interventions tailored towards the specific needs of the HNC patient [15]. The present study expands on previous research on exercise interventions in HNC, which have typically been initiated during active treatment, by implementing a combined exercise and education programme in the post-treatment phase [16–18]. Social deprivation, combined with high symptom burden, poor health literacy and social isolation, may impact how patients with head and neck cancer engage with rehabilitation [19, 20]. The inclusion of educational strategies in exercise programmes can improve patients’ understanding of the benefits, risks, and behavioural expectations of physical activity, thereby increasing exercise self-efficacy, a key predictor of sustained engagement with exercise in cancer survivors [21]. In addition, rehabilitation programmes that have input from a multi-disciplinary team may help to address the multi-faceted rehabilitative needs experienced by survivors of complex cancer. Existing models of rehabilitation for patients with complex cancers, which include health education delivered by a multi-disciplinary team, may be suitable for patients with head and neck cancer and require further investigation [22].

The aim of this study was to examine the feasibility and acceptability of a 10-week multi-modal exercise and education rehabilitation intervention to optimise physical function in patients post head and neck cancer treatment.

## Methods

This single-arm prospective study evaluated the feasibility and acceptability of an exercise and education rehabilitation programme for head and neck cancer survivors in a real-world, standard practice setting. The findings will inform the design, methodological considerations, and justification of a future adequately powered randomised controlled trial.

A convenience sample of patients under the care of maxillo-facial and ENT consultants in St James’s Hospital, a comprehensive cancer centre in Dublin, Ireland, was recruited between February and May 2025. Ethical approval was obtained through the St James’s Hospital and Tallaght University Hospital Research and Ethics Committee (Submission Number: 3872). The study is registered on ClinicalTrials.gov Registration Number: NCT06646861 (registered 17 October 2024). The study was conducted in accordance with the Declaration of Helsinki. The reporting standards for pilot/feasibility trials put forward by Consolidated Standards of Reporting Trials (CONSORT [23]) were followed in the preparation of this manuscript (Supplemental Material).

### Study population

Eligibility criteria included a signed consent form, aged over 18 years old, patients in the first 2 years post-treatment for HNC, and medically fit to participate in physical activity. Individuals with moderate or severe cognitive impairment, currently pregnant, or receiving treatment in the palliative setting were excluded from participation.

Members of the clinical team identified eligible persons from rehabilitation databases. The patient information leaflet and study invitation letter were mailed or emailed to patients entered on the database between mid-2023 and early 2025. Patients interested in participating were advised to contact the research team and were then invited to attend a screening assessment session to assess eligibility. Written and informed consent and baseline measures were then completed with participants.

### Intervention

The CaRe exercise programme was designed in accordance with international guidelines for best practice exercise prescription for people with cancer [5]. The programme was theoretically underpinned by the Theory of Planned Behaviour. The Theory of Planned Behaviour relates perceived behavioural control to self-efficacy [24]. The intervention used three techniques aimed to increase participants’ motivation to exercise including goal setting, instruction on exercise including information booklets, and regular feedback from the exercise professional (physiotherapist).

The exercise intervention included 10 weeks of twice weekly group-based online or in-person exercise sessions. Sessions were conducted under the supervision of a clinical physiotherapist with additional training in oncology who was not a part of the research study team. A choice of online and in-person sessions was offered as St James’s is a national centre, as participants lived over a large geographical area. Online sessions were offered through Zoom (Zoom Video Communications, Inc., 2020). Patient interest in each modality was examined to help determine the optimal intervention in future studies. Each exercise session lasted approximately 1 h and consisted of a combination of aerobic and resistance exercise.

***Aerobic exercise:*** included 20 to 30 min of moderate-intensity cardiovascular exercise using a variety of modalities such as walking or jogging on a treadmill, cycling, or rowing on a stationary ergometer for in-person participants and jogging, side-steps, and jumping jacks for online participants. Participants exercised to a target intensity of 12–15 on the BORG Rating of Perceived Exertion (RPE) scale.

***Resistance exercises:*** targeted the large muscle groups of the upper and lower extremities, were performed at 40% to 70% of the one repetition maximum (1-RM), and included two sets of 10–15 repetitions. When participants could complete the maximum number of repetitions for a given exercise, with good form, the resistance was increased (Robinson et al., 2024). Coloured elastic resistance bands (Theraband, Akron, OH) were given to patients for performing resistance exercises. Each colour represents a different band resistance. Eight major upper limb and lower limb muscle groups were exercised.

***Self-directed care:*** A home exercise programme was included as a self-managed component of the programme which aligns with national recommendations for survivorship care. This self-management component aimed to improve compliance and stimulate physical activity outside the exercise programme. Participants were encouraged to be moderately physically active for at least 30 min, three times per week in addition to the supervised programme. 

***Education programme:*** The exercise programme was supported with four 30-min education sessions. All participants were invited to attend these in-person sessions. The topics of the education sessions included the role of exercise in cancer rehabilitation (delivered by a registered physiotherapist), lymphoedema management in head and neck cancer (delivered by a lymphoedema therapist), management of cancer-related fatigue (delivered by an occupational therapist), and a question-and-answer session with a consultant maxillo-facial surgeon.

***Maintenance of exercise intervention:*** All patients completing the CaRe programme were offered an onward referral to a local community exercise setting.

### Outcomes

Outcomes were assessed at baseline (T1) and at the completion of the 10-week intervention (T2) by a research physiotherapist not involved in the provision of the intervention.

### Primary outcome

The primary outcome of this study was its feasibility aspects, including recruitment rates (percentage of eligible study population that consented to participation), programme adherence (number of prescribed supervised and unsupervised sessions completed), participant retention (percentage of patients that completed the programme), acceptability of the intervention, and adverse events. Reasons for attrition or non-compliance were identified through qualitative evaluation with participants.

### Secondary outcomes

The impact of the intervention on secondary measures was measured at two timepoints (pre- and post exercise intervention) including (1) neck pain: pte Neck Disability Index [25]; (2) quality of life: the functional assessment of cancer therapy head and neck questionnaire (FACT-HN) [26]; (3) physical function: the 30-s sit to stand test, hand grip strength, and Clinical Frailty Scale [26]; (4) self-reported physical activity: the Godin Leisure-Time Physical Activity Questionnaire [27]; (5) cancer-related fatigue: Brief Fatigue Inventory [28]; (6) oral intake: the Functional Oral Intake Scale [29]; and (7) lymphoedema: localised percentage water content using a lymph scanner (Delfin Technology). This is a non-invasive hand-held lymphoedema measurement device. It measures localised percentage water content (PWC) of tissue (2 anatomical points bilaterally).

### Qualitative evaluation

The qualitative element of the study was undertaken after completion of the exercise programme. Acceptability of the intervention was explored through semi-structured qualitative interviews. Semi-structured interview guides were developed by a researcher with experience in qualitative data collection (GS). Individual interviews were audio recorded and transcribed. Interviews lasted between 10 and 15 min and took place in-person from March to June 2025. Data were managed using NVivo 10 and analysed according to the thematic analysis approach described by Braun and Clarke [30].

### Statistical analysis

Participant demographics were described using frequencies for categorical variables, and minimum, maximum, mean, median, and standard deviation were reported for continuous variables.

Descriptive statistics were calculated for all secondary outcome variables at both pre- (T1) and post (T2) timepoints. Continuous variables were summarised using medians and interquartile ranges (IQR). Pre-post changes for each outcome were assessed using Wilcoxon signed-rank tests. Effect sizes for the Wilcoxon tests were calculated using the formula *r* = *Z*/√*N*, where *Z* is the test statistic and *N* is the number of paired observations for each variable, and interpreted following conventional benchmarks: small (*r* ≈ 0.1), medium (*r* ≈ 0.3), and large (*r* ≥ 0.5). As the analysis was exploratory, no adjustment for multiple comparisons was applied in the initial analysis. A Bonferroni-adjusted significance threshold was applied post hoc to identify which outcomes remained statistically significant after accounting for multiple testing.

All analyses were conducted using IBM SPSS Statistics version 29 (IBM Corp., Armonk, NY, USA). Missing data were handled via pairwise deletion; only participants with both T1 and T2 data for a given outcome were included in the corresponding Wilcoxon test. This meant that the number of cases per variable varied.

### Sample size

A formal power calculation was not appropriate for this feasibility study, as the primary objectives are to evaluate recruitment, retention, and procedural feasibility. A total sample of approximately 22–30 participants was set in keeping with norms in the literature, particularly when feasibility endpoints are the focus [31].

## Results

### Intervention feasibility

**Fig. 1:**
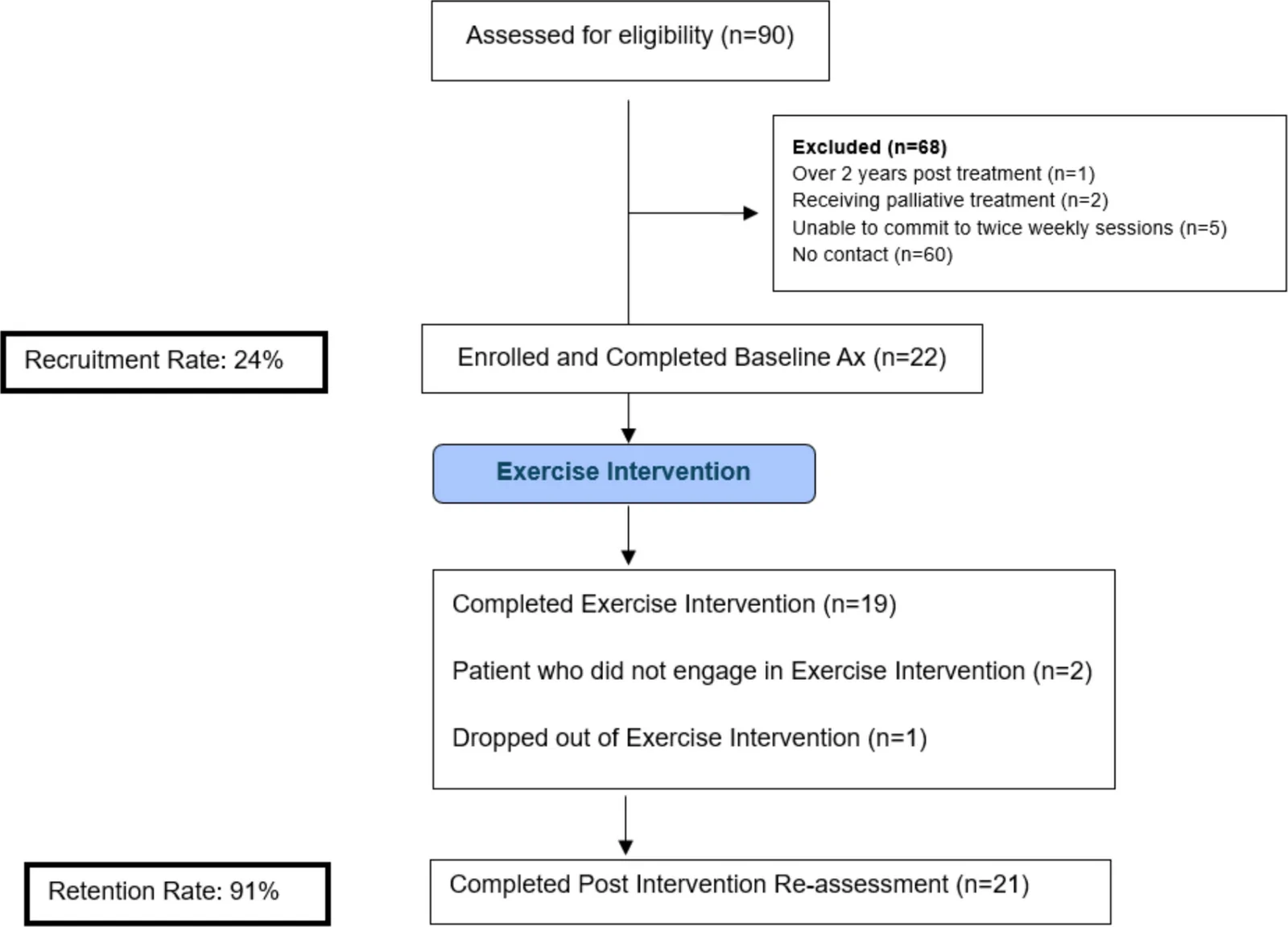
Flow of CaRe study participants

Recruitment letters were sent to 90 patients on the rehabilitation database for head and neck cancer. Twenty-eight patients responded to this letter (31%) and 22 people provided consent and attended an initial assessment (recruitment rate 24%) (Fig. 1).

In total, 36% (*n* = 8) of the study population were female, and most participants had a history of surgery and neck dissection and were on average 12 (SD 6.97) months post treatment (Table 1). One participant had an oropharyngeal primary tumour and was the only individual in the cohort with P16 expression associated with this tumour type (*n* = 1).

**Table 1:** Participant demographics

Characteristic	All participants (*n* = 22)
Age, years, mean (SD, range)	65.23 (14.53, 27–87)
Gender, % (*n*) Female Male	36.4 (8) 63.6 (14)
Highest education level, % (*n*) Primary school Junior/intercert Secondary school/leaving cert Primary degree Master’s degree	18.2 (4) 13.6 (3) 27.3 (6) 22.7 (5) 18.2 (4)
Smoking status, % (*n*) Current smoker Current vaper Ex-smoker Non-smoker	9.1 (2) 4.5 (1) 50 (11) 36.4 (8)
Tumour site, % (*n*) Oral cavity Other	82 (18) 18 (4)
Stage of disease, % (*n*) Stage 1 Stage 2 Stage 3 Stage 4	22.7 (5) 36.4 (8) 13.6 (3) 27.3 (6)
Cancer treatment, % (*n*) Surgery Radiation Chemotherapy Radioiodine	95.5 (21) 68.2 (15) 27.3 (6) 4.5 (1)
Surgical flap, % (*n*) Yes	31.8 (7)
Laryngectomy, % (*n*) Yes	4.5 (1)
Neck dissection, % (*n*) Unilateral Bilateral	77.3 (17) 18.2 (4)
Cancer treatment status Ongoing treatment, % (*n*) Mean months since end of treatment to start of study (SD)	4.5 (1) 12.33 (6.97)

**Fig. 2:**
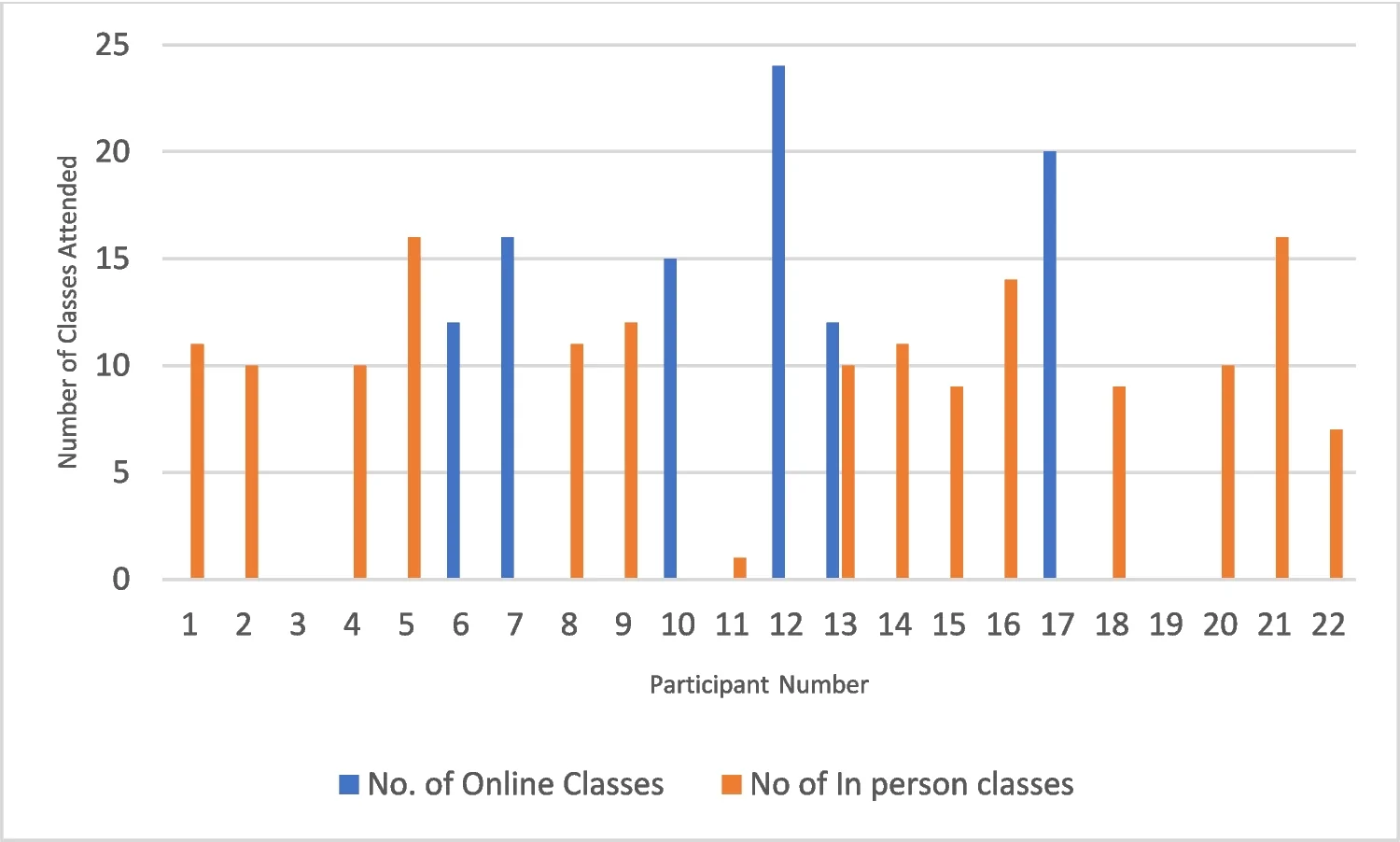
Exercise class attendance information

Five participants engaged with the online classes only (mean attendance, 16 classes; range 6–24). Most (*n* = 14) attended the in-person exercise classes (mean attendance, 10 classes; range 1–20). One person chose to do one online and one in-person class a week. Two participants were scheduled for the online programme however failed to log on to any classes despite two reminder calls from the research team. One participant attended for T2 re-assessment despite not engaging with the programme. One participant medically deteriorated after attending 1 class and dropped out of the intervention (Fig. 2).

Retention, defined as the percentage of participants still enrolled on the trial at the post-intervention assessment (T2), was 91%. Attendance at the in-person education sessions was 52% (the role of exercise in cancer rehabilitation (*n* = 9), lymphoedema management (*n* = 10), management of cancer-related fatigue (*n* = 12), and Q&A with surgeon (*n* = 12)). No serious adverse events occurred during the trial.

### Secondary outcomes

Over half (54.5%, *n* = 12) of participants screened positively for lymphoedema at T1. The same proportion were categorised as positive at T2; however, these were not the same participants each time. Eight participants were negative at both T1 and T2 and 10 participants were positive at both T1 and T2. Two participants changed from negative to positive and 2 participants changed from positive to negative. Lymph scanner measurements of the right cheek decreased significantly from T1 to T2 (Table 2).

**Table 2:** Summary table of outcome variables

Outcome	T1 median [IQR]	T2 median [IQR]	Number	*Z*	*p*	*r*
Weight, kg	77.0 [63.9–94.7]	75.0 [67.2–94.5]	21	0.052	0.959	0.01
R neck scanner (PWC)	45.5 [38.5–52.3]	45.4 [32.8–52.0]	21	0.523	0.601	0.11
L neck scanner (PWC)	41.0 [31.5–47.3]	40.5 [34.5–47.4]	21	0.322	0.748	0.07
**R cheek scanner (PWC)**	45.0 [41.0–47.0]	39.3 [23.5–43.9]	21	2.938	**0.003**	0.64
L cheek scanner (PWC)	40.5 [32.0–44.3]	34.9 [24.8–42.9]	21	1.912	0.056	0.42
**30-s STS, reps**	11.0 [10.0–14.0]	14.0 [11.5–14.5]	21	2.645	**0.008**	0.58
R Grip Strength, kg	28.65 [19.7–35.3]	28.66 [19.7–36.8]	19	0.682	0.496	0.16
L Grip Strength, kg	24.3 [19.7–30.8]	27.66 [18.2–35.4]	20	0.523	0.601	0.12
**CFS**	3.0 [3.0–4.0]	2.0 [1.0–2.5]	20	3.487	< 0.001*	0.78
**Oral Intake (FOIS)**	6.0 [5.0–6.3]	6.0 [6.0–7.0]	21	2.309	**0.021**	0.50
**PWB**	21.0 [17.0–26.0]	25.0 [22.0–26.0]	19	2.689	**0.007**	0.62
SWB	24.5 [21.3–27.8]	25.6 [21.0–28.0]	18	0.787	0.431	0.19
EWB	19.5 [18.0–23.8]	20.0 [19.0–24.0]	18	1.074	0.283	0.25
FWB	20.0 [15.5–28.0]	22.0 [18.0–28.0]	18	1.491	0.136	0.35
**HNCS**	27.0 [20.0–31.5]	32.0 [25.0–36.0]	18	3.296	< 0.001*	0.78
**FACT-HN total**	109.5 [95.5–124.0]	123.0 [114.0–133.8]	18	2.353	**0.019**	0.55
**Godin**	15.0 [5.75–28.0]	32.0 [19.5–42.8]	20	3.241	0.001*	0.72
**BFI**	2.8 [1.10–5.1]	1.09 [0.4–2.4]	20	2.577	**0.010**	0.58
NDI	6.5 [3.50–11.3]	6.0 [2.0–10.0]	20	0.214	0.831	0.05

*IQR*, interquartile range; *R*, right; *L*, left; *PWC*, percentage water content; *STS*, sit to stand; *CFS*, Clinical Frailty Scale; *FOIS*, Functional Oral Intake Scale; *PWB*, physical well-being; *SWB*, social well-being; *EWB*, emotional well-being; *FWB*, functional well-being; *HNCS*, head and neck cancer subscale; *FACT-HN*, Functional Assessment of Cancer Therapy–Head & Neck; *Godin*, Godin Leisure-Time Exercise Questionnaire; *BFI*, Brief Fatigue Inventory; *NDI*, Neck Disability Index

*Denotes variables that continue to show statistically significant differences between timepoints after Bonferroni correction is applied to *p*-values

Values in bold reached the predefined threshold for statistical significance

For functional outcomes, 30-s sit-to-stand repetitions increased at T2 while scores on the Clinical Frailty Scale decreased significantly with *n* = 15 (68%) participants showing reduced frailty at T2 (Table 2). For quality-of-life outcomes, physical well-being increased, and overall FACT-HN total quality-of-life scores improved (*p* = 0.019, Table 2). Head and neck cancer subscale scores improved significantly (*p* < 0.001). Self-reported physical activity increased, while cancer-related fatigue (BFI) decreased (Table 2).

Scores on the Functional Oral Intake Scale improved significantly from T1 to T2, with 8 participants reporting higher intake levels. Other outcomes such as weight, neck lymph scanner measures, grip strength, neck pain, and social/emotional and function well-being scores did not change significantly from T1 to T2.

### Patient feedback

Nineteen participants completed post-programme qualitative interviews. Three themes are presented in Table 3.

**Table 3:** Qualitative themes

**Positive aspects of the programme**	Motivation to exercise
	Shared or common purpose of group
	Managing side effects of treatment
	Option to engage online
	Guidance and supervision from exercise professionals
**Challenges engaging in programme**	Tailoring/individualising exercise to patients
	Commitment and effort is needed
	Uncertainty about starting exercise
	Group-based exercise
**Role of programme in recovery/survivorship**	Patients surprised by their own abilities
	Planning life after treatment
	Signified end of treatment

Participants reported many positive aspects to the exercise programme. This included increased motivation to exercise because of the structure of the programme. For some, exercising with others was a positive as it brought an element of competition, and for others it was the sense of comradery and sense of shared purpose in the class.

“You didn’t have to introduce yourself. Anybody who goes through this scenario…everybody knew they were there for the same reason, which is good, and it was very, very nice”. CaRe 05

“I found it very good…I think it was because I knew everybody else there had been sick. So I feel they understand, whereas I find other people don’t know” CaRe 15

Participants also valued the education sessions for the opportunity to talk with others:

“Those (education) classes…people were very chatty and there was a social dimension to it.” CaRe05

Many participants described the physical and psychological benefits of engaging in the exercise programme. This included improvements in fatigue, muscle strength, pain, and physical function.

“One big thing I noticed is when I’m in my stocking feet, say if I dropped a coin on the floor I can bend right down and pick it up. Where before I used to pull out a chair and go down on one knee to get down and pick it up.” CaRe 12

The guidance and support given by the exercise instructors were valued by participants:

“I think that motivates me to do something like coming somewhere from here and have someone that guides you and tells you what to do on the different equipment – that helps me.” CaRe15

Many participants were surprised by the benefits experienced during the programme:

“It surprised me how much it just got me walking that walk better and has built up my strength, taking away that lower back pain.” CaRe 10

“I sort of thought I was fit. But when I went to use them (machines) I didn’t feel I was as fit” CaRe 15

Some challenges associated with engaging in the programme centred on the effort involved in attending the programme twice weekly and the need to push yourself in the class:

“When you’re back at work, then it’s fitting in the days, fitting in the time. You could be in a meeting and then have to say, ‘I need to go.’” CaRe 06

“You can make the exercises a challenge. So if you watch his exercise and go faster than him (the instructor), it’s a challenge”. CaRe06

For many participants, the programme signified the end of their cancer treatment and provided an opportunity to consider their recovery and return to activity.

“You just finished the surgery and going into four different appointments and then you were gone. So it’s much easier phasing it – like now I’m definitely ready to be finished. I’m ready to move on to the next thing. And I think that this CaRe project helped me with that because it was an ending and I was working towards this ending…and I’m ready now to not forget about it, but you know, you have to move on.” CaRe13

“The instructors were very encouraging as in saying ‘You can do it, try it and do it and don’t let this be the end. Do not let the eight weeks just be the end of your fitness thing, keep going. That’s so helpful.” CaRe 12

## Discussion

This study demonstrated that a structured exercise and education intervention for patients following complex head and neck cancer treatment is feasible and acceptable. Measures of physical function and well-being all increased post intervention and engagement in the programme was high, with participants particularly valuing the social experience of the group programme. 

Passive recruitment methods resulted in a 24% uptake, which, although modest, reflects the challenges inherent in engaging this population. Participants were recruited following treatment; however, previous studies have reported greater recruitment but lower retention when patients are approached from the time of diagnosis [32]. Participants had undergone complex surgical procedures, including flap reconstructions and neck dissections, and were able to safely take part in group-based exercise sessions. Most participants opted for in-person sessions, reinforcing established preferences for direct supervision and peer support during exercise, particularly in female cohorts [33, 34]. The hybrid approach, which included in-person and remote options, was feasible, but participant feedback suggested that the social and motivational aspects of attending in-person sessions were particularly important. Many participants reported that the programme signified the end of their active cancer treatment and offered an opportunity to focus on the future. Participants attended the education sessions on topics they perceived as important to their recovery. This is consistent with previous findings that exercise interventions may serve as a meaningful transition point in survivorship, supporting patients’ adjustment to post-treatment life [35]. Participants valued supervision by an exercise specialist, which may reflect concerns about injury risk identified as a barrier to physical activity in previous studies [36]. Some participants reported that they would not have independently sought an exercise intervention following treatment, emphasising an opportunity for HCPs to provide more information and support regarding the role of physical activity in cancer care. Despite this, programme engagement was high, and participants provided positive feedback, particularly valuing the social experience of exercising alongside peers with similar cancer experiences. This highlights the potential psychosocial benefits of group-based interventions in this cohort, particularly given the visible and functional sequelae of head and neck cancer, which can contribute to social isolation and body-image concerns [37].

Results provide further support for both the psychosocial and physiological benefits of exercise in this population [15]. Physiologically, even with limited adherence, the intervention appeared to confer specific benefits in terms of reductions in frailty and improved muscle strength and overall physical well-being. Low skeletal muscle mass represents a negative prognostic factor for overall survival in patients affected by head and neck cancer undergoing radiotherapy or chemoradiotherapy [38, 39]. Often muscle impairments persist for years after therapy conclusion, highlighting the opportunity for interventions including resistance exercise to maximise muscle mass in this population [40]. Importantly, participants’ weight remained stable during the exercise intervention. This is particularly important in HNC, as unintentional weight loss can exacerbate treatment-related side effects and negatively impact clinical outcomes [41]. Median baseline physical activity scores indicated that most participants were initially sedentary or insufficiently active; however, the majority of participants increased their overall physical activity levels during the programme. This pattern of increased physical activity and strength alongside reduced frailty and stable body weight indicates the potential for meaningful functional gains with post treatment exercise interventions.

The observed benefits, combined with positive participant feedback, raise the question of whether such interventions should be offered as standard care. The intervention was delivered in close collaboration with a multi-disciplinary team (MDT), which was critical for addressing the complex needs of this patient group. The prevalence of lymphoedema and cancer-related fatigue in the group highlights the need for coordinated care. Future work, including randomised controlled trials, is warranted to determine efficacy, optimal delivery models, and long-term outcomes, as well as to explore strategies for improving engagement among those who remain less active or do not experience fatigue improvement.

### Strengths and limitations

Important strengths of the intervention include the application of the programme in a real-world clinical practice setting. Secondly, we consider the timing of the intervention to be advantageous. This time period, termed the “recovery or rehabilitation period”, may be the optimal window for commencing an exercise programme to reverse a downward trajectory in activity levels and fitness as well as addressing any lingering treatment-related side effects such as fatigue and less helpful weight changes [42].

Although several outcomes showed statistically significant improvements, these findings should be interpreted as preliminary. The analyses were exploratory, with increased risk of type I error due to multiple comparisons. The primary purpose of this feasibility study was to test study procedures. The results of the feasibility study may be used for optimisation of the intervention and may serve as a basis for a larger definitive trial. In future trials, structured monitoring of home-based exercise would allow for a more comprehensive assessment of intervention fidelity and dose response relationships**.**

## Conclusion

The exercise intervention was achievable, safe, and acceptable, improving physical activity, strength, frailty, and well-being. Group-based, in-person sessions provided valued psychosocial support, supporting exercise as a survivorship transition point following complex treatment completion period.

## Supplementary Information

Below is the link to the electronic supplementary material.

ESM 1 (DOC 225 KB)

## Data Availability

The datasets generated during and/or analysed during the current study are available from the corresponding author on reasonable request.

## Declarations

### Data

No new data are associated with this article.
